# Different Susceptibility of Mammalian Cell Lines to Severe Fever with Thrombocytopenia Syndrome Virus Infection

**DOI:** 10.3390/v17101380

**Published:** 2025-10-16

**Authors:** Marla Anggita, Samuel Nyampong, Weiyin Hu, Hiroshi Shimoda, Daisuke Hayasaka

**Affiliations:** 1 Laboratory of Veterinary Microbiology, Joint Graduate School of Veterinary Medicine, Yamaguchi University, Yamaguchi 753-8515, Japan; marla.anggita@ugm.ac.id (M.A.); c502tnw@yamaguchi-u.ac.jp (S.N.); c010tnw@yamaguchi-u.ac.jp (W.H.); hshimoda@yamaguchi-u.ac.jp (H.S.); 2 Laboratory of Microbiology, Faculty of Veterinary Medicine, Gadjah Mada University, Yogyakarta 55281, Indonesia; 3 Laboratory of Veterinary Microbiology, Joint Faculty of Veterinary Medicine, Yamaguchi University, Yamaguchi 753-8515, Japan; 4 Division of Pathogenic Microorganisms, Research Center for Thermotolerant Microbial Resources, Yamaguchi University, Yamaguchi 753-8515, Japan

**Keywords:** SFTSV, cytopathic effect, viral replication, plaque-forming assay, cell lines

## Abstract

Severe Fever with Thrombocytopenia Syndrome (SFTS) is an emerging tick-borne infectious disease that poses a significant public health threat. SFTS virus (SFTSV) has a broad host range, including humans, cats, and natural reservoir species. Therefore, cultured cell lines derived from different mammalian species are useful for understanding the susceptibility of SFTSV in hosts. In this study, we evaluated pathogenicity and infectivity, focusing on cytopathic effect (CPE) induction and growth kinetics of SFTSV in several mammalian cell lines, including our original tiger-derived TLT, wild deer–derived DFKT and DFLT, and hedgehog-derived HHoVT. Following SFTSV infection, TLT, CRFK (cat), FCWF-4 (cat), and CPK (porcine) cells exhibited CPE, whereas Vero E6 (monkey), A549 (human), BHK-21 (hamster), DFKT, DFLT, and HHoVT cells did not. Infectious viral yields in the supernatants of TLT, CRFK, FCWF-4, Vero E6, and BHK-21 were higher than those of CPK, A549, DFLT, and DFKT. SFTSV infection in hedgehog-derived HHoVT cells was very limited. These observations suggest that features such as viral CPE and virus yield following SFTSV infection depend on cell type. It is noteworthy that TLT formed clear plaques that were easy to count, indicating that TLT cells are useful for the titration of infectious SFTSV by plaque-forming assay. Our results provide useful information and tools for further elucidating the mechanisms of SFTSV infectivity, proliferation, and pathogenicity using in vitro models.

## 1. Introduction

Severe Fever with Thrombocytopenia Syndrome (SFTS) is an emerging tick-borne disease caused by the SFTS virus (SFTSV), which is currently classified within the order *Bunyavirales*, family *Phenuiviridae*, genus *Bandavirus*, and species *Dabie bandavirus* [1]. SFTSV was first reported in China and has been identified in Japan, South Korea, Taiwan, Vietnam, Thailand, Myanmar and Pakistan [2,3,4,5,6,7,8,9]. SFTSV is mainly transmitted to humans by ticks such as *Haemaphysalis longicornis* and *Amblyomma testudinarium* [3,10,11]. Human-to-human transmission has also been reported through direct exposure to infected blood or bodily fluids [12,13].

Clinical manifestations of SFTS include high fever, decreased platelet and white blood cell counts (thrombocytopenia and leukopenia), and gastrointestinal symptoms such as vomiting, nausea, and diarrhea [14]. In severe cases, the disease may lead to multi-organ failure with mortality rates of up to 30% [13,15]. Despite its significant public health impact, the pathogenesis of SFTS remains incompletely understood, and neither vaccines nor specific antiviral therapies are currently available.

SFTSV has been maintained in nature through amplifying hosts and small mammals such as rodents and shrews as reservoirs [16,17]. SFTSV infections have been identified in various animals such as wild boars, wild deer, cattle, pigs, goats, cheetahs, dogs, and cats [18,19,20,21,22,23,24,25,26]. In particular, many cat cases of SFTS have been reported in Japan, and SFTSV infections from animals to humans have been reported, suggesting that SFTS is an important public health concern as an emerging zoonotic disease [27,28,29].

In vitro models using cultured cells have been used as a crucial tool to elucidate the infectivity, pathogenicity, viral tropism and host–pathogen interactions of pathogenic viruses. SFTSV demonstrates variable infectivity across various cell types derived from human and animals [30]. Human cell lines derived from various organs such as lung, liver, and kidney are susceptible to SFTSV infection [31]. Diverse animal cell lines such as Vero, COS7 [32], DH82 (canine macrophage-like) and L929 (murine fibroblasts) cells are also permissive to SFTSV infection [3]. Infectious susceptibility of SFTSV depends on cell type; for example, lymphocyte cell lines showed lower susceptibility to SFTSV than adherent cell lines [32]. Also, cytopathic effects (CPE) due to SFTSV infection appear to depend on cell type [33,34].

An infectious cell culture system is also essential to quantify the SFTSV titer. Viral titrations have been achieved through various methodologies such as plaque-forming assay (PFA), focus-forming assay (FFA), tissue culture infectious dose (TCID_50_), immunofluorescence assay (IFA), and quantitative reverse transcription polymerase chain reaction (qRT-PCR) [2,35,36,37]. In general, FFA is preferred for titrating infectious SFTSV, because SFTSV infection often resulted in limited CPE in most cell lines [38]. FFA provides enhanced sensitivity through antibody-based detection in infected cells; however, the results depend on the quality of the antibody, and the methods are generally time-consuming and costly compared with PFA [39,40].

The differential susceptibility patterns observed among various mammalian cells suggest that cellular factors play crucial roles in determining infection outcomes such as viral replication efficiency and CPE appearance [34,41,42]. Therefore, the selection of appropriate cell lines and the establishment of in vitro models are fundamental procedures to elucidate the properties of SFTSV infectivity and pathogenicity.

This study aims to systematically evaluate and compare susceptibility to SFTSV infection in multiple mammalian cell lines, including our original cell lines derived from tiger, wild deer, and hedgehog. Our results will provide updated information on useful cell cultures for in vitro SFTSV studies to enhance our understanding of SFTSV cellular tropism.

## 2. Materials and Methods

### 2.1. Cells and Virus

Vero E6 (African green monkey kidney) and BHK-21 (baby hamster kidney-21) cells were kindly provided by Dr. Hiroaki Kariwa, Hokkaido University, and have been maintained in our laboratory. FCWF-4 (feline fetal macrophage), CRFK (Crandell-Rees feline kidney), A549 (human alveolar basal epithelial), and CPK (porcine kidney) cell lines were obtained from the American Type Culture Collection (ATCC). Mammalian cell lines used in this study are listed in Table 1. TLT, DFKT, DFLT, and HHoVT cells were originally established in our laboratory from tiger liver, wild deer kidney, wild deer liver, and hedgehog ovary, respectively [43]. Briefly, the cells were immortalized by introducing Large T antigen of simian virus 40 and have cultivated more than 40 passages [43]. All cells were maintained in Dulbecco’s Modified Eagle Medium (DMEM) (Gibco, Life Technologies Corporation, Grand Island, NY, USA) supplemented with 10% fetal bovine serum (FBS) (Sigma-Aldrich, Co., St. Louis, MO, USA). The SFTSV Tk-F123 and Ng-F264 strains isolated from SFTS cases [44] were propagated in Vero E6 cells, and the stock viruses were stored at −80 °C until use. All experiments involving infectious SFTSV were conducted in a Biosafety Level-3 facility at Yamaguchi University, Japan, in accordance with institutional biosafety guidelines and national regulations.

### 2.2. FFA of SFTSV

FFA was conducted according to the methods referred to a previous study [35]. Briefly, Vero E6 cells were seeded into 96-well plates and inoculated with two-fold serial dilutions of SFTSV. Following a 90 min incubation at 37 °C, the SFTSV-infected cells were overlaid with 1% methylcellulose (MC) DMEM with 2% FBS and incubated at 37 °C with 5% CO_2_ for three days. After fixation with 4% paraformaldehyde, viral foci were detected using anti-SFTSV nucleoprotein monoclonal antibodies [45], followed by treatment with a goat anti-mouse IgG conjugated with horseradish peroxidase American Qualex International, Inc., San Clemente, CA, USA). Visualization of viral foci was performed by adding a substrate mixture of 3,3′-diaminobenzidine (Fujifilm Wako Pure Chemical Corp., Osaka, Japan) in distilled water along with 0.1% hydrogen peroxide (Wako Pure Chemical Industries, Ltd., Osaka, Japan). Viral titers were measured as focus-forming units per milliliter (FFU/mL).

### 2.3. SFTSV Infections in Cell Lines

Each cell line was seeded in 24-well plates at a density of 5 × 10^4^ to 1 × 10^5^ cells/well and incubated at 37 °C with 5% CO_2_ until they reached confluence. The confluent cells were inoculated with SFTSV Tk-F123 at a multiplicity of infection (MOI) of 0.01. The formation of CPE was observed daily. The supernatants were collected daily from 0 to 7 days post-infection (dpi). Collected supernatants were subjected to viral titration using FFA.

### 2.4. PFA of SFTSV

PFA was performed using CRFK, FCWF-4, and TLT cells. Cells were seeded in 24-well plates in DMEM supplemented with 10% FBS and were incubated until a confluent monolayer was formed. Ten-fold serial dilutions of SFTSV in DMEM with 2% FBS were inoculated onto confluent cells. Following a 90 min incubation, DMEM with 2% FBS containing 0.5%, 1%, or 2% MC were overlaid and incubated at 37 °C with 5% CO_2_. At 5 to 7 dpi, cells were fixed with 4% paraformaldehyde, and plaque formation was observed by staining the cells with 0.1% crystal violet. The virus titer was determined as plaque-forming unit per milliliter (PFU/mL).

### 2.5. Real-Time RT-PCR

Real-time RT-PCR was performed to the methods referred to our previous study [35]. Briefly, SFTSV viral RNA was extracted using ISOGEN-LS (NIPPON GENE Co., Ltd., Toyama, Japan). Primers of 965F (5′-GCRAGGAGCAACAARCAAACATC-3′) and 1069R (5′-GCCTGAGTCGGTCTTGATGT C-3′), and probe FAM/5′-CTCCCRCCC-3′/ZEN/5′-TGGCTACCAAAGC-3′ (Integrated DNA Technologies, Inc., Shinjuku, Japan) were used and real-time reactions were carried out using a TaKaRa One Step PrimeScript™ RT-PCR Perfect Real Time Kit (Takara Bio Inc., Shiga, Japan). Reactions were performed at 42 °C for 5 min, 40 cycles at 95 °C for 10 s, at 95 °C for 5 s, and at 60 °C for 34 s. The viral copy numbers were determined as the ratio of the copy numbers to the standard control prepared from an SFTSV gene-cloned plasmid vector [35].

## 3. Results

### 3.1. CPE Formation in SFTSV-Infected Cell Lines

**Figure 1:**
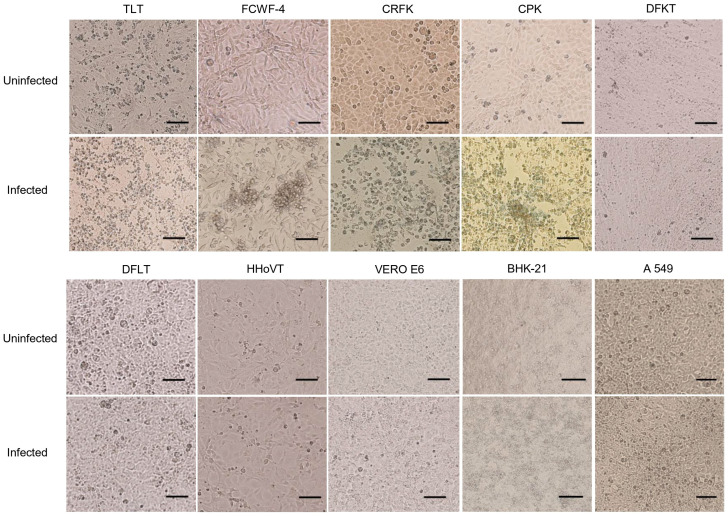
CPE formation in different cell lines following SFTSV infection. Each cell line was infected with SFTSV at an MOI of 0.01 and microscopic images of TLT (3 dpi), FCWF-4 (5 dpi), CRFK (5 dpi), CPK (4 dpi), DFKT (5 dpi), DFLT (5 dpi), HHoVT (5 dpi), Vero E6 (5 dpi), BHK-21 (5 dpi), and A549 (5 dpi) are shown. SFTSV-infected TLT, FCWF-4, CRFK, and CPK cells exhibit apparent CPE compared with uninfected controls. Morphologies of SFTSV-infected DFKT, DFLT, HHoVT, Vero E6, BHK-21, and A549 cells are similar to those of uninfected controls. Scale bars indicate 100 μm.

We first focused on CPE appearance in each cell line following SFTSV infection. TLT (tiger), FCWF-4 (cat), CRFK (cat), and CPK (pig) cells clearly showed CPE at 3, 5, 5, and 4 dpi, respectively (Figure 1 and Table 1). CPE features include cell rounding, aggregation, detachment, and shrinkage in size (Figure 1).

On the other hand, DFKT (wild deer), DFLT (wild deer), HHoVT (hedgehog), Vero E6 (monkey), BHK-21 (hamster), and A549 (human) cells did not exhibit apparent CPE after SFTSV infection (Figure 1). These observations suggest that CPE formation due to SFTSV infection depends on cell species, and Felidae (cat- and tiger-derived) cells exhibited CPE.

### 3.2. Growth Kinetics of SFTSV in Cell Lines

**Figure 2:**
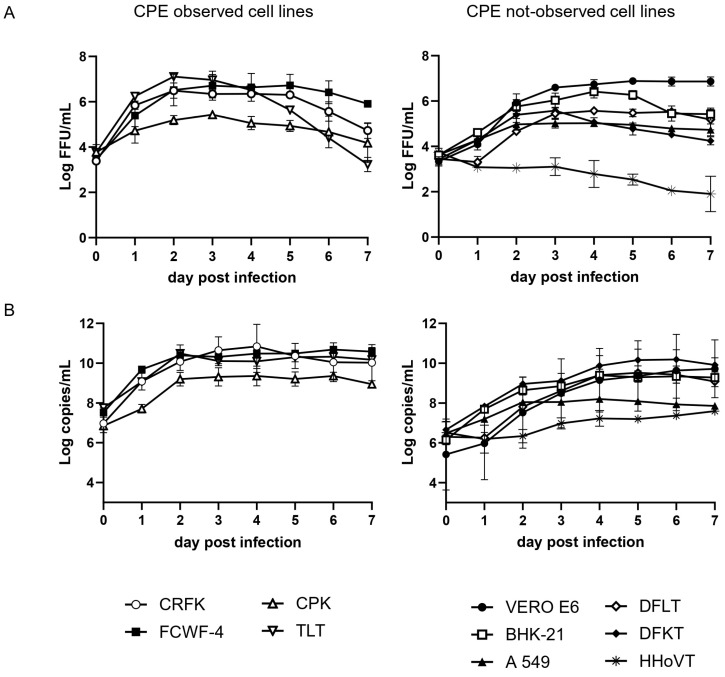
Growth kinetics of SFTSV in different cell lines. (**A**) Infectious virus titers and (**B**) viral copy numbers in the cultured supernatants of each cell. Each cell line was infected with SFTSV at an MOI of 0.01, and the supernatants were collected daily. Data are presented as the mean ± standard deviation from three independent experiments.

We next examined the growth kinetics of SFTSV in each cell line and determined the viral titers in the supernatants. In CPE-observed cell lines TLT, FCWF-4, CRFK, and CPK, viral titers increased until around 2 dpi and then were maintained or decreased (Figure 2A). Interestingly, TLT, FCWF-4, and CRFK showed higher virus yields in the supernatants, indicating 10^3.3^, 10^3.1^, and 10^3.1^ times increases at 2 dpi compared with those of 0 dpi, respectively (Table 1 and Figure 2A). CPK exhibited comparatively lower virus yield with 10^1.4^ times increase at 2 dpi compared with that of 0 dpi (Table 1 and Figure 2A). In these CPE-observed cells, viral titers of supernatants decreased after CPE-appeared days in TLT, FCWF-4, CRFK, and CPK cells at 3, 5, 5, and 4 dpi, respectively (Figure 2A). Also, the viral RNA levels were not significantly increased or decreased after CPE (Figure 2B), implying that viral yields did not occur after CPE in these cells.

In non-CPE cell lines of DFKT, DFLT, Vero E6, BHK-21, and A549, viral titers in the supernatants increased until 3–4 dpi (Figure 2A). Interestingly, viral titers in the supernatants varied among cell lines, showing 10^1.4^ to 10^4.0^ -fold increases at 4 dpi compared with those of 0 dpi (Table 1 and Figure 2A). In these cells, infectious viral titers in the supernatants were almost maintained or slightly decreased by 7 dpi (Figure 2A). On the other hand, viral RNA levels of Vero E6, BHK-21, DFLT, and DFKT continued to increase until around 6 dpi, whereas the levels of A549 decreased after 4 dpi (Figure 2B). This discrepancy might be due to offsetting titer decreases at 37 °C, because our unpublished results showed that infectious titers of SFTSV infectivity at 37 °C were gradually decreased (approximately 10^0.2^ FFU per day) [46]. Therefore, these observations suggest that viral propagation in these cells was maintained for 6–7 days.

On the other hand, viral titers of the supernatant in HHoVT cells did not increase over 0 dpi (Figure 2A). Viral RNA levels of HHoVT cells were slightly increased by 7 dpi; however, these levels were significantly lower than those of other cell lines (Figure 2B), suggesting that infectivity and viral propagation of SFTSV were very low in HHoVT cells.

### 3.3. PFA of SFTSV

**Figure 3:**
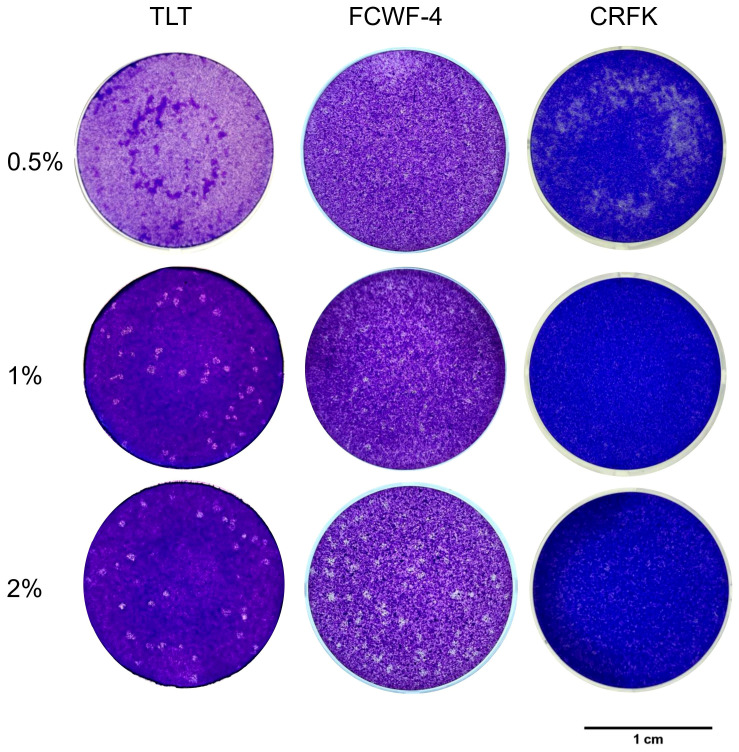
Plaque formation of SFTSV-infected TLT, FCWF-4, and CRFK cells. After inoculation with SFTSV, cells were overlaid with medium containing 0.5%, 1%, or 2% MC. The plaques were observed after 5 days in TLT cells and 7 days on FCWF-4 cells. FCWF-4 cells exhibited limited visibility of plaques compared with TLT cells. CRFK cells showed no visible plaques. Plaque morphologies with 1% MC were clear, whereas 2% MC reduced plaque size and visibility.

We next performed PFA using CPE-observed cell lines TLT, CRFK, and FCWF-4. Interestingly, TLT exhibited clear plaque formation with 1% and 2% MC medium, but not 0.5% MC (Figure 3). On the other hand, FCWF-4 cells showed plaques, but the plaques were difficult to count due to variable sizes and non-round morphology (Figure 3). CRFK did not form apparent plaques under any MC condition (Figure 3). From these results, TLT is likely to be useful for the PFA of SFTSV. Therefore, we compared the titers of SFTSV stock viruses (Table 2). Titers of PFU were approximately 2–3 times higher than FFU, but these differences were not significant as virus titers were in the log phase. These results indicate that TLT cells are useful for the titration of SFTSV by PFA depending on the experimental design.

## 4. Discussion

In this study, we evaluated the susceptibility to SFTSV in several mammalian cell lines, including our original cell lines. Tiger-derived TLT, cat-derived CRFK, and FCWF-4 cells exhibited apparent CPE and high viral yields following SFTSV infection. Pig-derived CPK cells also showed CPE, but viral yields were comparatively lower than those of TLT, CRFK, and FCWF-4 cells. Monkey-derived Vero E6, hamster-derived BHK-21, human-derived A549, and wild deer-derived DFKT and DFLT cells did not exhibit CPE. Among them, Vero E6 and BHK-21 showed comparatively higher viral yields than other cell lines. SFTSV infection in hedgehog-derived HHoVT cells was very limited. These observations suggest that different features, including CPE and virus yields, following SFTSV infection depend on the cell type. Our results provide useful information and tools for elucidating the mechanism of SFTSV infectivity, proliferation, and pathogenicity using in vitro models.

CPE induction in virus-infected cells is one of the keys to elucidating the pathogenic mechanism of infectious viruses. Previous studies have stated that viruses have complex life cycles in host cells and affect many aspects depending on the host’s immune response and replication mechanisms of the virus [47]. Viral infection could cause damage to the cells and affect viral replication by hijacking their cellular processes for its own replication [48]. CPE due to viral infection is generally discerned through cellular responses such as necrosis and apoptosis [49,50]. Although SFTSV infection causes severe disease in humans and some kinds of animals, the type of cell death and the mechanism of CPE due to SFTSV are not fully elucidated. To elucidate SFTSV pathogenesis, it is important to provide a useful tool for the analysis of CPE in vitro. In this study, we identified four CPE-induced cell lines-TLT, CRFK, FCWF-4, and CPK-and these cell lines are expected to be useful to elucidate the mechanisms of CPE induced by SFTSV infection in vitro.

Our results indicated that the levels of viral propagation varied depending on cell type, including higher yields (TLT, CRFK, FCWF-4, Vero E6, and BHK-21) with more than about 10^3^ fold and lower yields (CPK, A549, DFLT and DFKT) with less than about 10^2^-fold increases in FFU compared with those of 0 dpi at around the peaks. High virus replication in Vero and BHK cells also aligns with previous findings [32]. Interestingly, among higher yield cells, TLT, CRFK, and FCWF-4 cells were CPE-induced, while Vero E6 and BHK-21 did not exhibit CPE. In addition, CPK cells showed CPE, but viral yields were comparatively low, suggesting that CPE formation is not simply related to high viral propagation. Our findings provide useful information and tools to examine the mechanism of viral replication and proliferation of SFTSV in vitro by comparing cell lines that exhibit higher and lower virus propagation.

SFTS cases have been reported not only in humans but also in several animal species, including cheetahs, cats, and dogs [22,24,51]. In particular, there are many SFTS cat cases in Japan [20,25,26], and it seems that family *Felidae*, including cats, are susceptible to SFTSV infection and can result in severe disease, because fatality of SFTS in cats was up to 60% and two fatal cases of cheetah were reported [24,25,51]. Previous studies of in vivo experiments and positive cases in cat species revealed severe lesions in the spleen, lymph nodes, and gastrointestinal tract, suggesting a significant impact of SFTSV on felines [25,51,52]. For one of the mechanisms of SFTSV pathogenicity in mammalian cells, the anti-STAT2 activity of SFTSV has been shown to determine species-specific pathogenicity in hosts cell [53]. STAT2 is involved in type I and type III interferon signaling, which are important for the immune response against viral infection [54,55]. The inability of SFTSV NSs protein to bind to STAT1 and STAT2 of murine cells makes murine cells permissive to SFTSV infection [53], and the mechanism involving STAT1 in humans and cats during SFTSV infection may be different and might affect the pathogenicity of SFTSV [56].

Therefore, cell lines including cats and other *Felidae* are required as tools for further analyses of SFTSV infection in vitro. In this study, tiger-derived TLT, cat-derived CRFK and FCWF-4 cells were very susceptible to SFTSV infection with high viral propagation and CPE appearance, suggesting that these cells are useful to further elucidate the high pathogenicity of SFTSV in cats and related animals. In particular, our original cell line TLT showed high viral propagation and induced apparent CPE, including clear plaque formation; thus, further studies using this TLT cell line will provide useful clues to elucidate the mechanism of SFTSV infectivity and pathogenicity.

SFTSV infects multiple kinds of animals, including wildlife and livestock [21,23], although those animals are unlikely to develop diseases [18,27]. For example, SFTSV surveillance on wild deer in Japan conducted between 2010 and 2020 showed that 55% of deer tested positive for anti-SFTSV antibodies, and 25% during 2013–2014, indicating notable seroprevalence among wild deer [22,57]. A study conducted in Korea detected SFTSV viral RNA in water deer tissues, one of the most common wild ungulates in Korea, thus raising concern about transmission between humans and wildlife [58]. In this study, we showed the susceptibility to SFTSV of our original wild deer-derived cells DFKT and DFLT, and these cell lines may be useful models to examine SFTSV infectivity and pathogenicity in deer.

European hedgehogs are suggested to be reservoirs of some viral diseases such as tick-borne encephalitis virus (TBEV) and coronaviruses [59,60,61]. However, our original hedgehog-derived HHoVT cells did not show apparent viral infectivity or propagation. In preliminary experiments, HHoVT cells did not exhibit susceptibility to SFTSV infection even at higher MOI (0.1 and 1). From the current data, we cannot conclude the factors of non-infectivity of SFTSV in hedgehogs; however, it seems important to elucidate the mechanism of inability of infection in this mammalian cell. Although SFTSV infectivity in HHoVT cells was limited, this cell line seems to be useful to determine a key of SFTSV infectivity in mammalian cells, focusing on receptors or other cell factors by specific gene knockout or overexpression experiments.

For titration of infectious SFTSV, immunostaining methods such as FFA are required, because SFTSV infection does not induce CPE in most cell lines [3,56,62]. However, FFA is generally time-consuming and costly compared with PFA. Taniguchi et al. reported a PFA method for neutralization assay using highly passaged SFTSV strain [36]; however, the cell lines utilized for PFA for SFTSV have been limited. In this study, we successfully achieved plaque formation using TLT and FCWF-4 cells, and TLT showed clear plaques that were easy to count. We used medium including methylcellulose to make foci, and methylcellulose offers better safety characteristics and handling convenience within containment compared to agar, even though agar provides proven performance and cost-effectiveness [63]. The titers of PFU by PFA were not significantly different compared with those of FFU by FFA in log phase. Our results provide useful information and a cell line for the PFA for SFTSV.

## 5. Conclusions

This study presents a comprehensive analysis of cell line susceptibility to SFTSV infection, and our findings will provide useful information and tools for further elucidation of the mechanisms of SFTSV infectivity, proliferation, and pathogenicity using in vitro models. In particular, our original tiger-derived TLT cell line is expected to support further analyses of the SFTSV high pathogenicity in vitro and serves as a useful cell for PFA. Although SFTSV susceptibility in our original cell line of hedgehog-derived HHoVT cell line was limited, this cell line is expected to be useful for determining the factors of SFTSV infectivity as a negative control compared with susceptible mammalian cells. Our results will contribute to further studies of SFTSV, including elucidation of the mechanisms of infectivity and pathogenicity in vitro, as well as for the development of improved diagnostic and research tools and the development of strategies to combat this emerging viral threat.

## Figures and Tables

**Table 1:** Cytopathic effect formation in SFTSV-infected cell lines.

	Origin			CPE Observed	Viral Titers (FFU/mL)	
	Species	Tissue				
CPE cells				dpi	0 dpi	2 dpi
TLT	Tiger	Liver	established	3	10^3.8^	10^7.1^
FCWF-4	Cat	Macrophage	commercial	5	10^3.4^	10^6.5^
CRFK	Cat	Kidney	commercial	5	10^3.4^	10^6.5^
CPK	Pig	Kidney	commercial	4	10^3.8^	10^5.2^
Non-CPE cells					0 dpi	4 dpi
DFKT	Wild deer	Kidney	established	-	10^3.4^	10^5.2^
DFLT	Wild deer	Liver	established	-	10^3.4^	10^5.6^
HHoVT	Hedgehog	Ovary	established	-	10^3.4^	-
Vero E6	Monkey	Kidney	commercial	-	10^3.4^	10^6.7^
BHK-21	Hamster	Kidney	commercial	-	10^3.4^	10^6.4^
A549	Human	Lung	commercial	-	10^3.4^	10^5.0^

**Table 2:** Comparison of SFTSV viral titration of FFA and PFA.

SFTSV Strain	FFA (FFU/mL)	PFA (PFU/mL)
	Vero E6	TLT
Tk-F123	4.3 × 10^7^	7.3 × 10^7^
Ng-F264	4.8 × 10^7^	1.3 × 10^8^

## Data Availability

The raw data supporting the conclusions of this article will be made available by the authors on request.
